# Evaluation of bowel wall flow by color Doppler ultrasound in the assessment of inflammatory bowel disease activity in pediatric patients

**DOI:** 10.1590/0100-3984.2023.0039-en

**Published:** 2023

**Authors:** Marco Aurélio Castellano, Vanessa Scheeffer, Vanessa Petersen, Themis Reverbel da Silveira

**Affiliations:** 1 Hospital da Criança Santo Antônio da Santa Casa de Misericórdia de Porto Alegre, Porto Alegre, RS, Brazil; 2 Hospital Moinhos de Vento, Porto Alegre, RS, Brazil; 3 Universidade Federal de Ciências da Saúde de Porto Alegre (UFCSPA), Porto Alegre, RS, Brazil

**Keywords:** Inflammatory bowel diseases, Leukocyte L1 antigen complex, Crohn disease, Colitis, ulcerative, Ultrasonography, Doenças inflamatórias intestinais, Complexo antígeno L1 leucocitário, Doença de Crohn, Colite ulcerativa, Ultrassonografia

## Abstract

**Objective:**

To assess inflammatory bowel disease (IBD) activity with Doppler ultrasound in pediatric patients, comparing the accuracy of the ultrasound findings with that of the concentrations of fecal calprotectin (FC).

**Materials and Methods:**

In a consecutive series, we evaluated 53 examinations of 44 pediatric patients seen between 2014 and 2020: 28 with Crohn’s disease, 15 with ulcerative colitis, and one with IBD unclassified. The diagnosis of IBD was made in accordance with the Porto criteria. The alteration studied in the greatest detail was bowel wall flow, which was classified by the lead investigator and two pediatric radiologists, all of whom were blinded to the FC concentrations and the other ultrasound findings. Bowel wall flow was categorized as low if there were up to 2 Doppler ultrasound signals/cm^2^, moderate if there were 3-5 signals/cm^2^, and high if there were more than 5 signals/cm^2^.

**Results:**

The agreement among the radiologists was substantial (kappa = 0.73). In cases in which ultrasound showed low bowel wall flow, the median FC concentration was 92 µg/g (interquartile range, 33-661 µg/g), whereas it was 2,286 µg/g (interquartile range, 1,728-5,612 µg/g) in those in which ultrasound showed high bowel wall flow. In the sample as a whole, the sensitivity and specificity of ultrasound was 89.7% and 92.0%, respectively, for the detection of inflammatory activity; 95.5% and 90.9%, respectively, for the detection of Crohn’s disease; and 81.3% and 100.0%, respectively, for the detection of ulcerative colitis.

**Conclusion:**

Ultrasound of the bowel wall showed a strong correlation with FC concentrations in the assessment of inflammatory activity in pediatric patients with IBD.

## Abstract (pt)

**Objetivo:**

Avaliar a atividade da doença inflamatória intestinal (DII) por ultrassonografia (US) com Doppler em cores, comparada à concentração de calprotectina fecal (CF) em pacientes pediátricos.

**Materiais e Métodos:**

Em uma série consecutiva, no período entre 2014 e 2020, foram avaliados 53 exames de 44 pacientes pediátricos: 28 casos de doença de Crohn, 15 de colite ulcerativa e um de colite indeterminada. O diagnóstico da DII foi feito pelos critérios de Porto. O fluxo parietal foi a alteração estudada mais detalhadamente e classificada pelo pesquisador principal e por dois radiologistas pediátricos cegados aos valores de CF e de US Doppler. Baixo fluxo parietal foi definido pela captação de até 2 sinais de US Doppler/cm^2^, fluxo moderado entre 3 e 5 sinais/cm^2^ e alto fluxo mais de 5 sinais/cm^2^.

**Resultados:**

Houve concordância substancial entre os radiologistas (kappa = 0,73). Nos exames com baixo fluxo parietal a CF média foi 92 µg/g (intervalo interquartil: 33-661 µg/g) e nos exames com alto fluxo a CF média foi 2.286 µg/g (intervalo interquartil: 1.728-5.612 µg/g). Na amostra total, a US demonstrou sensibilidade de 89,7% e especificidade de 92,0% para detecção da atividade inflamatória, 95,5% e 90,9% na doença de Crohn e 81,3% e 100,0% na colite ulcerativa, respectivamente.

**Conclusão:**

Houve forte correlação entre a US da parede intestinal e os valores da concentração de CF na avaliação da atividade inflamatória na DII de pacientes pediátricos.

## INTRODUCTION

Inflammatory bowel disease (IBD) is a progressive disease characterized by chronic inflammation of the gastrointestinal tract, being divided into Crohn’s disease, ulcerative colitis, and IBD unclassified. In Western countries, the prevalence and incidence of IBD have increased in recent decades, patients under 18 years of age accounting for up to 25% of cases^(^1^)^.

The pediatric phenotypes of IBD are more aggressive than are those of IBD in adults^(^2^)^. Colonoscopy with biopsy is not always feasible in the pediatric population, because it is a complex procedure that requires anesthesia and the participation of a trained specialist. Noninvasive markers of disease activity are central to assessing IBD activity for clinical decision making. Measurement of fecal calprotectin (FC) is the most widely used noninvasive method for assessing IBD activity^(^3^)^. Bunn et al.^(^4^)^ showed that FC levels correlate strongly with endoscopic and histological activity scores in children with IBD. In patients without warning symptoms, a negative FC result can avoid costly invasive procedures. In this context, imaging tests play a central role in the diagnosis of IBD, the follow-up of patients with IBD, and the management of IBD complications. In a study examining the diagnostic accuracy of transabdominal ultrasound for intestinal inflammation in children with IBD, van Wassenaer et al.^(^5^)^ found a sensitivity and specificity of up to 93% for ultrasound in comparison with colonoscopy and magnetic resonance enterography (MRE). Other studies have compared Doppler ultrasound and MRE in terms of their accuracy for assessing IBD activity in pediatric patients^(^6^,^7^)^. Although there is good agreement between MRE and Doppler ultrasound regarding disease location and activity^(^6^)^, MRE is more costly; requires a specialized center, prolonged fasting, and long test times; and is limited to use in older children because the need for anesthesia is a contraindication to distending the bowel with fluid^(^7^-^9^)^.

Ultrasound is a universally accepted, noninvasive, low-cost method that uses no ionizing radiation. It can be a valuable tool in the assessment of IBD activity in clinical practice, color Doppler ultrasound being particularly useful in that setting.

In the present study, we sought to evaluate the accuracy of Doppler ultrasound in assessing disease activity in pediatric patients with IBD, comparing it with that of FC measurement.

## MATERIALS AND METHODS

This was a prospective, cross-sectional noninterventional study of consecutive patients seen between November 2014 and December 2020. We evaluated 53 examinations of 44 pediatric patients, comparing Doppler ultrasound findings and FC concentrations. In nine patients, Doppler ultrasound findings and FC concentrations were evaluated at two different time points.

For children ≤ 7 years of age, the parents or legal guardians gave written informed consent. Children > 7 years of age gave written informed assent. The study was approved by the local research ethics committee (CAEE no. 80543417.90000.5683).

The inclusion criteria for the study were: patients under 18 years of age and diagnosed with IBD according to the Porto criteria^(^10^)^. The exclusion criteria were: patients with a history and clinical data of chronic digestive diseases (food allergies, neoplastic conditions, celiac disease, eosinophilic colitis/enteritis or irritable bowel syndrome). (food allergies, neoplastic conditions, celiac disease, eosinophilic colitis/enteritis, or irritable bowel syndrome) were excluded. The exclusion criteria for an ultrasound examination were as follows: having extensive abdominal scarring; not having fasted before the procedure; and presenting with severe obesity (defined as a body mass index above the 95th percentile). The interval between FC measurements and Doppler ultrasound examinations did not exceed 14 days. Of a total of 50 patients, six were excluded, for the following reasons: obesity, in two; extensive fibrosis of the abdominal wall, in one; and inappropriate stool sample collection, in three.

The clinical manifestations of IBD vary depending on factors such as the intestinal segment involved, the extent of involvement, and the duration of disease. Abdominal pain and diarrhea are the most common symptoms, seen in 50-90% of patients. Perianal fistulas are more common in patients with Crohn’s disease (particularly in those with severe disease), whereas rectal bleeding is more common in patients with ulcerative colitis.

In children with IBD, reduced intestinal absorption can lead to nutritional changes and, consequently, impaired growth and development. Extraintestinal manifestations can occur, including the following: hepatic manifestations (autoimmune hepatitis and primary sclerosing cholangitis); dermatological manifestations (erythema nodosum and pyoderma gangrenosum); ophthalmological manifestations (uveitis and iritis); hematological manifestations (anemia and thromboembolism); and musculoskeletal manifestations (arthritis, arthralgia, osteopenia, and ankylosing spondylitis, as well as changes in growth rate and pubertal development). The Pediatric Crohn’s Disease Activity Index and the Pediatric Ulcerative Colitis Activity Index are clinical disease activity indices that are used in order to assess the severity of IBD; guide the follow-up of patients; and evaluate the therapeutic response. They are multiple-item scores based on symptoms, laboratory test results, clinical examination, and growth assessment, classifying disease activity as absent, mild, moderate, or severe.

Initial laboratory tests include a complete blood count, including platelets, liver enzymes (aspartate aminotransferase and alanine aminotransferase), bilirubins, amylase, urea, creatinine, and nonspecific markers of inflammation, such as erythrocyte sedimentation rate and C-reactive protein. Serological markers of IBD activity include anti-*Saccharomyces cerevisiae* antibodies (mainly for Crohn’s disease) and perinuclear antineutrophil cytoplasmic antibodies (mainly for ulcerative colitis). The presence of leukocyte-derived proteins (particularly calprotectin) in stool is a highly sensitive intestinal marker of IBD and plays a central role in the diagnosis and follow-up of IBD, the FC test being recommended by the European Society for Paediatric Gastroenterology, Hepatology and Nutrition. Concentrations of FC > 250 µg/g indicate active disease.

### Abdominal color Doppler ultrasound

All patients were examined by a pediatric radiologist with more than 15 years of experience in the field. The following were analyzed: bowel wall flow, bowel wall thickness, bowel wall stratification, and peristalsis. Mesenteric fat echogenicity, lymph node enlargement, fluid collections, and free fluid in the abdomen were also investigated.

The lead investigator performed all ultrasound examinations, selecting the images that showed bowel wall flow. Subsequently, the lead investigator and two other pediatric radiologists, all of whom were blinded to the FC concentrations and the other ultrasound findings, independently reviewed the images, answering the following questions: 1) Can you identify color Doppler flow signals on the images? ( ) Yes ( ) No; 2) If you checked Yes, how would you classify the case, in accordance with the criteria described by Spalinger et al.^(^11^)^? a) low flow: ≤ 2 color Doppler signals/cm^2^; b) moderate flow: 3-5 color Doppler signals/cm^2^; or c) high flow: > 5 color Doppler signals/cm^2^; and 3) How many color Doppler signals can you identify in the box with the highest blood flow?

Doppler ultrasound was performed with 3-12 MHz convex and linear transducers (HD11XE; Philips Healthcare, Best, the Netherlands), without the use of contrast media. With a partially full bladder, patients were initially examined with a low-frequency convex transducer, for abdominal organ evaluation as well as to provide a panoramic view of the pelvis and rectosigmoid. They were subsequently examined with a high-frequency linear transducer for large bowel evaluation in the axial and longitudinal planes, starting from the rectosigmoid. In a counterclockwise direction, the descending colon, transverse colon, ascending colon, and cecum were examined. Subsequently, the small bowel was examined in the longitudinal and axial planes. The right iliac fossa was gradually compressed in order to identify the ileocecal junction, the iliac vessels and the psoas muscle being used as landmarks. The abdomen was divided into four quadrants, and the small bowel loops were examined by the lawnmower scanning approach described by Elliot et al.^(^12^)^.

Bowel loop thickness was measured from the serosa to the mucosa, in triplicate, by gray-scale ultrasound with a linear transducer, the highest of the three measurements being selected. In a recent systematic review and meta-analysis describing bowel wall thickness in healthy children, van Wassernaer et al. reported an upper limit of 1.9 mm in the small intestine and colon^(^13^)^. In the present study, bowel wall thicknesses > 2 mm in the small intestine and colon were considered abnormal.

Color Doppler ultrasound was performed with the use of a low wall filter, as well as the highest possible color gain and the lowest possible pulse repetition frequency, to avoid flow artifacts and aliasing. In the segment showing the greatest bowel wall thickness, color Doppler ultrasound was performed with the use of a 1-2 cm^2^ area of interest, in accordance with Spalinger et al.^(^11^)^, and the number of signals was counted. This determination served as an estimate of inflammatory hyperemia, which was classified as low (≤ 2 Doppler signals/cm^2^), moderate (3-5 Doppler signals/cm^2^), or high (> 5 Doppler signals/cm^2^).

### FC

On the day of FC measurement, first morning stool samples were collected in the homes of the patients with the use of a stool collection kit. Samples collected one day before measurement were refrigerated. An enzyme immunoassay was used in order to extract and quantify FC in accordance with the manufacturer instructions (ELISA Calprotectin; Phadia Laboratory Systems - Thermo Fisher Scientific, Waltham, MA, USA). Inflammatory activity was defined as an FC concentration > 250 µg/g^(^14^)^.

### Statistical analysis

Continuous variables are expressed as mean and standard deviation or as median and interquartile range for non-normally distributed data. Categorical variables are expressed as counts and percentages. The level of agreement among the radiologists was assessed by the weighted kappa statistic, and the correlation between FC concentrations and color Doppler ultrasound findings was assessed by Spearman’s correlation coefficient. The ability of Doppler ultrasound to show inflammatory activity was expressed as sensitivity, specificity, and likelihood ratios, with their respective 95% confidence intervals. Additional between-group comparisons were performed by signed rank tests. Values of *p* < 0.05 were considered statistically significant. The data were processed and analyzed with the IBM SPSS Statistics software package, version 22.0 (IBM Corporation, Armonk, NY, USA).

## RESULTS

A total of 44 IBD patients ≤ 18 years of age were included in the study. The mean age was 12.9 ± 3.8 years, and most of the patients were > 10 years of age. All of the patients were White, and 24 (54.5%) were male. Crohn’s disease was the most common type of IBD (in 63.6%). Of the 28 patients with Crohn’s disease, 18 (64.3%) were male. Of the 15 patients with ulcerative colitis, 10 (66.7%) were female. Only one patient had IBD unclassified, and that patient was male. Table 1 shows the demographic and clinical data.

**Table 1:** Demographic and clinical characteristics of the patients.

Characteristic	(N = 44)
Age (years), n (%)	
< 4	2 (4.5)
4-10	7 (15.9)
> 10	35 (79.5)
Mean ± standard deviation	12.9 ± 3.8
Minimum-maximum	0.6-17.9
Sex, n (%)	
Male	24 (54.5)
Female	20 (45.5)
Type of IBD, n (%)	
Crohn’s disease	28 (63.6)
Ulcerative colitis	15 (34.1)
IBD unclassified	1 ( 2.3)

A total of 53 FC measurements were performed. The median FC concentration was 997 µg/g (IQR, 217-1,897 µg/g). Of the 53 FC measurements, 10 showed concentrations ranging from < 50 µg/g to 100 µg/g, four showed concentrations ranging from 100 µg/g to 250 µg/g and 39 showed concentrations > 250 µg/g. The lowest FC concentration was 9 µg/g, and the highest was 6,000 µg/g.

**Figure 1:**
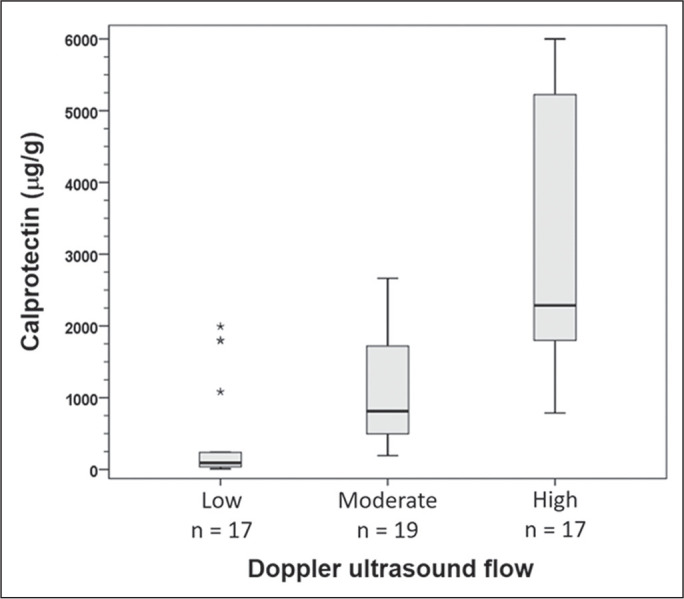
Correlation between FC concentrations and bowel wall flow on color Doppler ultrasound (Spearman’s correlation coefficient = 0.72).

There was substantial agreement among the three radiologists regarding the classification of Doppler ultrasound findings (weighted kappa = 0.73). Blood flow to the bowel wall, as assessed by color Doppler ultrasound (Figure 1), was classified as low (≤ 2 Doppler signals/cm^2^) in 17 of the 53 examinations, moderate (3-5 Doppler signals/cm^2^) in 19, and high (> 5 Doppler signals/cm^2^) in 17.

**Figure 2:**
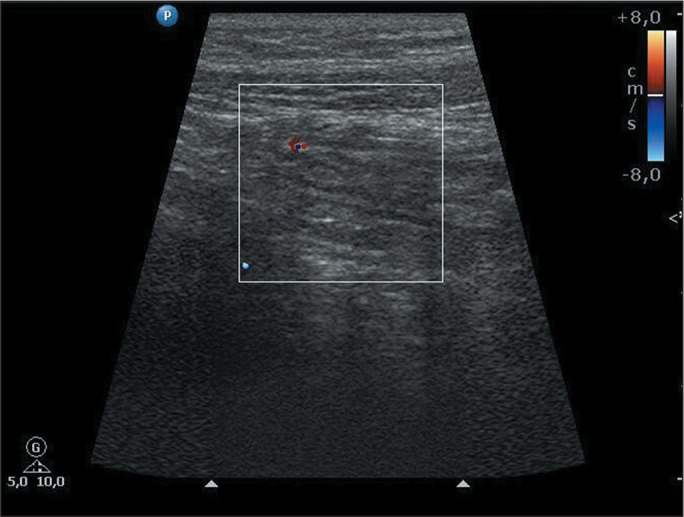
Color Doppler ultrasound image of a 9-year-old female patient with Crohn’s disease, showing mild inflammatory activity in the ileum, as evidenced by minimal bowel wall flow. FC concentration, 9.9 µg/g.

**Figure 3:**
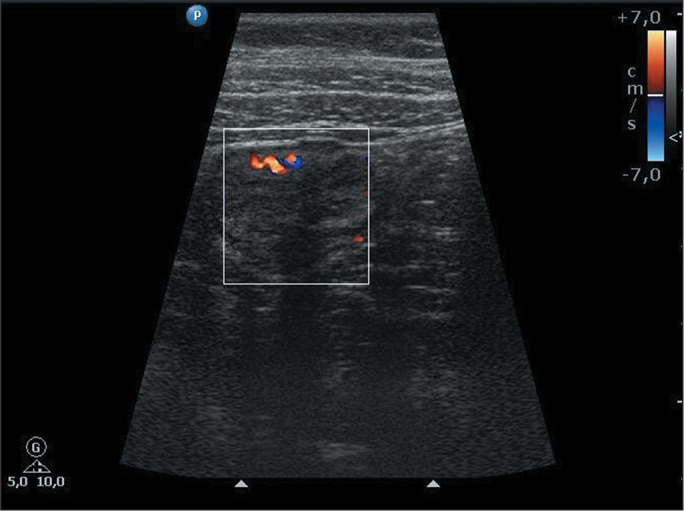
Color Doppler ultrasound image of a 12-year-old female patient with ulcerative colitis, showing moderate inflammatory activity in the descending colon, as evidenced by the presence of 3-5 Doppler signals/cm^2^ in the bowel wall. FC concentration, 1,733 µg/g.

**Figure 4:**
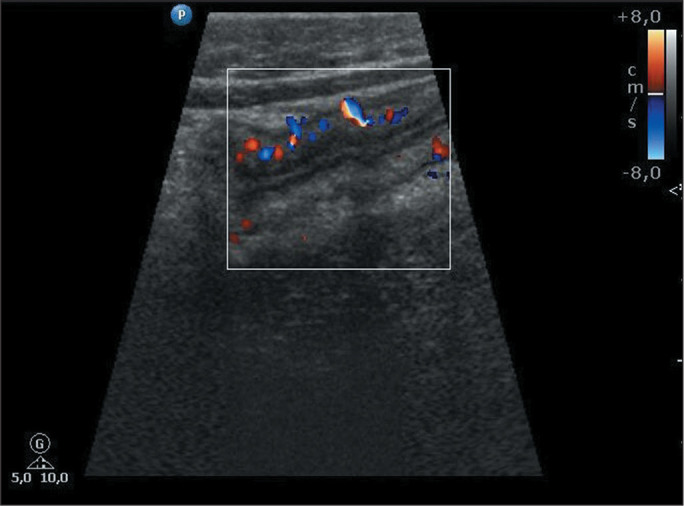
Color Doppler ultrasound image of a 13-year-old male patient with Crohn’s disease, showing severe inflammatory activity in the ileum, as evidenced by a high bowel wall flow. FC concentration, 1,800 µg/g.

There was a strong correlation between Doppler ultrasound findings and FC concentrations (r_s_ = 0.72; *p* < 0.001). In cases in which bowel wall flow was classified as low (≤ 2 Doppler signals/cm^2^), the median FC concentration was 92 µg/g (IQR, 33-661 µg/g). In cases in which bowel wall flow was classified as high (> 5 Doppler signals/cm^2^), the median FC concentration was 2,286 µg/g (IQR, 1,728-5,612 µg/g). Figures 2, 3, and 4 exemplify the various degrees of bowel wall flow.

When compared with FC concentrations, Doppler ultrasound findings, in the sample as a whole, showed a sensitivity of 89.7% (95% CI, 75.8-97.1) and a specificity of 92.9% (95% CI, 66.1-99.8) for the detection of IBD activity. The positive and negative likelihood ratios were 12.6 (95% CI, 1.9-38.3) and 0.11 (95% CI, 0.04-0.28), respectively. In the patients with Crohn’s disease, the sensitivity and specificity of Doppler ultrasound were 95.5% and 90.9%, respectively. In the patients with ulcerative colitis, the sensitivity and specificity of Doppler ultrasound was 81.3% and 100.0%, respectively (Table 2).

**Table 2:** Accuracy of Doppler ultrasound in the assessment of inflammatory activity in pediatric patients with IBD.

Disease	Sensitivity % (95% CI)	Specificity % (95% CI)	Positive likelihood ratio % (95% CI)	Negative likelihood ratio % (95% CI)
IBD	(n = 39) 89.7 (75.8-97.1)	(n = 14) 92.9 (66.1-99.8)	12.56 (1.89-83.30)	0.11 (0.04-0.28)
Crohn’s disease	(n = 22) 95.5 (77.2-99.9)	(n = 11) 90.1 (58.7-99.8)	10.50 (1.62-68.19)	0.05 (0.01-0.34)
Ulcerative colitis	(n = 16) 81.3 (54.3-96.0)	(n = 3) 100.0 (29.2-100.0)	4.84 (0.45-72.55)	0.19 (0.07-0.60)

Concentrations of FC were associated with bowel wall thickness values. In the patients with FC concentrations < 250 µg/g, the median bowel wall thickness was 1.85 mm (IQR, 1.30-2.40 mm), whereas it was 4.2 mm (IQR, 3.15-5 mm) in those with FC concentrations > 250 µg/g.

## DISCUSSION

This was a prospective study designed to compare Doppler ultrasound findings and FC concentrations in pediatric patients with IBD. We observed a strong correlation between high FC levels and bowel wall flow for inflammatory activity in pediatric patients with IBD.

The European Society for Paediatric Gastroenterology, Hepatology and Nutrition recommends that FC concentrations be measured as a marker of inflammatory activity in pediatric patients with IBD^(^15^)^. Because the concentration of calprotectin is approximately six times higher in feces than in serum, the FC test is useful in patients with bowel disease^(^16^)^. Calprotectin is a complex protein that accounts for 60% of the cytoplasmic proteins found in human neutrophils, being found in lower concentrations in monocytes and macrophages. It has fungicidal and bactericidal activities^(^17^)^, being resistant to intestinal proteolysis. The concentration of calprotectin in feces reflects neutrophil migration through the inflamed mucosa, and calprotectin is eliminated in the feces during active inflammation. In a systematic review and meta-analysis, FC was shown to be more sensitive than C-reactive protein and fecal lactoferrin as a noninvasive biomarker of inflammatory activity^(^18^)^. Various studies have shown that high concentrations of FC correlate with measurements of inflammatory activity in the colon and small intestine^(^4^,^19^)^. It has been suggested that concentrations > 250 µg/g are indicative of active disease^(^14^,^19^)^. More recently, Haisma et al. reported that FC concentrations > 250 µg/g can be a prognostic indicator^(^20^)^.

Studies have shown an association between Doppler ultrasound analysis of bowel wall flow and endoscopic analysis of inflammatory activity. In a meta-analysis of 1,558 adult patients, ultrasound was found to have high diagnostic accuracy for active Crohn’s disease^(^21^)^. Novak et al. reported that ultrasound can replace endoscopy in guiding management of the disease in adults^(^22^)^. However, studies in pediatric patients are rare^(^23^)^. Fodor et al.^(^24^)^ evaluated the performance of abdominal ultrasound in monitoring 30 children with ulcerative colitis, comparing ultrasound with FC measurement and colonoscopy. The authors showed that abdominal ultrasound findings correlated well with colonoscopy findings and with high FC levels. Dolinger et al.^(^25^)^ investigated the usefulness of small bowel ultrasound in the evaluation of an early response to treatment with infliximab in 13 pediatric patients with Crohn’s disease and reported that hyperemia is the first parameter to be affected.

Our study is original in that it compares ultrasound and FC measurement in the assessment of IBD activity. According to the European Society for Paediatric Gastroenterology, Hepatology and Nutrition, ultrasound is a valuable tool in the follow-up of patients with IBD. Because ultrasound is accurate and provides immediate results, it is currently used at our institution for rapid clinical decision-making in the evaluation of inflammatory activity.

Kellar et al. have recently developed a new score to evaluate pediatric IBD^(^26^)^. The authors considered bowel wall thickness and mesenteric inflammatory fat the most important ultrasound parameters. Although they described the presence of hyperemia, they did not quantify bowel wall flow and categorized bowel wall thickness ≤ 3.9 mm as normal. Unlike those authors, we found that the median bowel wall thickness was 4.2 mm among the patients with FC concentrations > 250 µg/g, which we considered to be indicative of active disease. In addition, when comparing bowel wall flow and FC concentrations, we found the former to have high sensitivity and specificity, as well as a strong correlation between bowel wall thickness and FC concentrations. A thicker bowel wall translates to a higher FC concentration. In patients with FC concentrations < 250 µg/g, the median bowel wall thickness was < 2 mm, whereas it was 4.2 mm in those with FC concentrations > 250 µg/g.

Our study has some limitations. First, it was a single-center study. Second, the study sample was relatively small and heterogeneous. Third and most important, Doppler ultrasound findings and FC concentrations were not compared with endoscopic findings. However, our results show that it is possible to establish a correlation between bowel wall flow and FC levels.

## CONCLUSION

Ultrasound proved useful in assessing IBD activity, correlating well with FC concentrations.
